# The role of C-peptide in diabetes and its complications: an updated review

**DOI:** 10.3389/fendo.2023.1256093

**Published:** 2023-09-07

**Authors:** Jintao Chen, Yajing Huang, Chuanfeng Liu, Jingwei Chi, Yangang Wang, Lili Xu

**Affiliations:** Department of Endocrinology, The Affiliated Hospital of Qingdao University, Qingdao, China

**Keywords:** C-peptide, diabetic kidney disease, diabetic retinopathy, diabetic peripheral neuropathy, non-alcoholic fatty liver disease, macrovascular complications

## Abstract

Worldwide, diabetes and its complications have seriously affected people’s quality of life and become a serious public health problem. C-peptide is not only an indicator of pancreatic β-cell function, but also a biologically active peptide that can bind to cell membrane surface signaling molecules and activate downstream signaling pathways to play antioxidant, anti-apoptotic and inflammatory roles, or regulate cellular transcription through internalization. It is complex how C-peptide is related to diabetic complications. Both deficiencies and overproduction can lead to complications, but their mechanisms of action may be different. C-peptide replacement therapy has shown beneficial effects on diabetic complications in animal models when C-peptide is deficient, but results from clinical trials have been unsatisfactory. The complex pattern of the relationship between C-peptide and diabetic chronic complications has not yet been fully understood. Future basic and clinical studies of C-peptide replacement therapies will need to focus on baseline levels of C-peptide in addition to more attention also needs to be paid to post-treatment C-peptide levels to explore the optimal range of fasting C-peptide and postprandial C-peptide maintenance.

## 1 Introduction

Globally, the incidence of diabetes is rapidly increasing, affecting almost 537million people, with approximately 109.6 million in China (1–3). Under hyperglycemic conditions, protein and lipid glycosylation, overproduction of reactive oxygen species (ROS), tissue expression of pro-inflammatory factors, and damage to vascular endothelium all promote the occurrence of diabetic complications (4). As diabetes progresses, a variety of complications occur, such as retinopathy, kidney disease, neuropathy, cardiovascular disease, and so forth. The complications of diabetes have become a serious global health issue that significantly impacts people’s quality of life (5) The leading cause of end-stage renal disease is diabetic kidney disease (DKD), as diabetes prevalence increases, the global incidence of end-stage kidney disease is on the rise (6). Diabetes is a leading cause of death due to myocardial infarction, and The risk of coronary heart disease in diabetic patients without a history of coronary disease is similar to that in non-diabetic patients with a history of coronary disease (7). Current clinical trials are increasingly focused on the impact of novel hypoglycemic drugs on cardiovascular events and renal prognosis, and the focus of diabetes management has shifted from merely controlling blood glucose to impacting cardiovascular and renal prognosis. However, there is still no definitive treatment to reverse diabetic complications. Even if diabetic patients are given intensified glycemic control at an early stage, the occurrence of complications is still unavoidable. Therefore, finding treatments to prevent and reverse diabetic complications is a key focus of the comprehensive management of type 2 diabetes mellitus (T2DM). As beta cells secrete C-peptide and insulin at equimolar levels, C-peptide can serve as an indicator of beta-cell reserve function. In the years following C-peptide’s discovery, the traditional view is that its biological activity promotes the folding of proinsulin within beta cells. However, according to research evidence collected over the past 20 years, C-peptide is also a biologically active peptide that may influence diabetic complications (8). A normal result of a C-peptide test ranges from 0.5 ng/mL to 2.0 ng/mL (or 0.17 to 0.83 nmol/L), which may differ slightly from lab to lab. C-peptide can bind with cell membrane surface signaling molecules, activate downstream signaling pathways, play anti-oxidative, anti-apoptotic roles, regulate inflammatory responses, or regulate cellular transcription through internalization (9). In type 1 diabetes mellitus (T1DM), C-peptide and insulin are deficient, and progressive beta-cell dysfunction can also be observed in the late stage of T2DM. Evidence from animal and *in vitro* experiments shows that when C-peptide is deficient, C-peptide replacement therapy can improve renal lesions (10), retinopathy (11), and peripheral neuropathy (12) by exerting anti-inflammatory, anti-apoptotic, and anti-oxidative effects. In the early stages of T2DM, C-peptide secretion is higher than physiological concentration, and C-peptide can also exert a pro-inflammatory effect, damaging vascular endothelium, and promoting vascular lesion occurrence (13, 14). On one hand, further research is needed to determine the relationship between C-peptide and diabetic complications. Both deficiency and over-secretion of C-peptide can promote the onset of diabetic complications, but the underlying mechanisms might differ. On the other hand, the biological activity of C-peptide can be a new breakthrough for the treatment and prevention of diabetic complications. As a result, we have summarized the current research progress on the relationship between C-peptide and diabetic complications as follows:

## 2 Physiological functions of C-peptide

### 2.1 C-peptide and cell signal transduction

Some studies indicate that C-peptide can function as a classical peptide hormone, binding to G-protein-coupled receptors on the cell surface to initiate cascading cellular signal transduction reactions (15). However, other research suggests that C-peptide also has some non-classical intracellular functions. C-peptide demonstrates a specific binding affinity towards the cell membrane, thereby initiating intracellular signal transduction via G-protein receptors and Ca^2+^-dependent pathways. This process consequently enhances the activity of endothelial nitric oxide synthase (eNOS), Na^+^/K^+^/ATPase, and phosphatidylinositol-3 kinase (PI-3-K). Furthermore, it activates the mitogen-activated protein kinases (MAPK) pathway and augments the activation and expression of particular transcription factors. These outcomes hold significant implications for anti-inflammatory, anti-oxidative, and cellular protective mechanisms (16). The signaling pathways involved in C-peptide mainly include: Ca^2+^ dependent pathway, p38 mitogen-activated protein kinase, extracellular signal-regulated kinase-1/2 (Erk-1/2), Akt phosphorylation, and production of endothelial cell nitric oxide (NO) (17, 18).

Lindahl’s research indicates that C-peptide can be internalized in target cells. C-peptide is internalized and located in the cytoplasm of Swiss 3T3 and HEK-293 cells and can bind with cytoplasmic proteins. Labeling revealed that C-peptide is transported into the cell nucleus (19). Subsequent investigations revealed that C-peptide is capable of associating with histones present in the nucleolus, thereby enhancing the acetylation at the lysine residue 16 of histone H4 located in the promoter region of ribosomal RNA genes. This particular interaction facilitates the transcription of genes responsible for encoding ribosomal RNA (20).

The cellular biology of C-peptide is largely unknown, especially concerning the peptide’s pathway across the cell membrane for internalization. Research by Luppi and colleagues demonstrated the co-localization of C-peptide with early endosome markers that support endocytosis (8). The pathway of C-peptide internalization is still in need of further exploration, and understanding its internalization pathways can further assist us in understanding the mechanisms through which it exerts intracellular activity.

### 2.2 Antioxidant and anti-apoptotic effects of C-peptide

Current research evidence on endothelial cells suggests that C-peptide can directly reduce the generation of ROS (21). C-peptide exhibits the ability to influence the nicotinamide adenine dinucleotide phosphate (NADPH) oxidase that is activated on the plasma membrane by high glucose levels. It achieves this by curtailing excessive ROS production through the restoration of mitochondrial electron transport chain activity within endothelial cells (22, 23). Research by Bhatt and colleagues indicates that C-peptide can activate AMP-activated protein kinase-α (AMPK-α), an enzyme that can reduce the production of NADPH and ROS in mitochondria and endothelial cell apoptosis (23). As AMPK-α plays a core role in energy metabolism and diabetes, this target could have broad therapeutic implications.

Animal experiments have demonstrated that C-peptide can reduce cellular apoptosis in the aorta, heart, and kidneys of diabetic mice by downregulating the activation of transglutaminase 2 (TGase2), an apoptosis-promoting enzyme mediated by ROS, in endothelial cells exposed to a high-glucose environment (24). Studies conducted *in vitro* have demonstrated that the supplementation of C-peptide exogenously has the capacity to diminish apoptosis in pancreatic islet cells induced by high glucose concentrations (25)..

C-peptide can downregulate the activation of transcription factor p53 and mitochondrial adaptor protein P66 caused by high glucose memory, thus reducing the apoptosis of vascular endothelial cells. Exogenous supplementation with C-peptide can prevent vascular endothelial damage caused by high glucose memory (26).

Conditions characterized by prolonged high glucose and elevated ROS levels may lead to a reduction in insulin gene expression. This is potentially mediated through the downregulation of transcription factors such as pancreatic duodenal homeobox 1 (PDX-1) and musculoaponeurotic fibrosarcoma oncogene family A (MafA) (27). Furthermore, such conditions may also accelerate the rate of cell apoptosis.

### 2.3 C-peptide and inflammatory response

C-peptide at physiological concentrations has anti-inflammatory effects, but some reports also suggest that C-peptide has pro-inflammatory effects, which may be related to its over-secretion. C-peptide has been observed to significantly attenuate the secretion of inflammatory factors, including IL-6, IL-8, MIP-1α, and MIP-1β, in U-937 monocytes under conditions of high glucose stimulation. Additionally, it decreases their adhesion to human aortic endothelial cells. This could potentially be attributed to the inhibition of excessive ROS production, thereby reducing the activation of nuclear factor-κB (NF-κB) (28). C-peptide also has the potential to lower the expression of adhesion molecules, such as P-selectin and intercellular adhesion molecule 1, in rat mesenteric arteries. As a result, the interaction between leukocytes and endothelial cells is diminished (29).

Clinical research has uncovered that in obese patients suffering from type 2 diabetes, there exists a positive correlation between the concentration of C-peptide and pro-inflammatory chemokine ligand-2 (C-C chemokine ligand -2, CCL2), as well as E-selectin. Conversely, a negative correlation has been observed between C-peptide concentration and the anti-inflammatory factor IL-10 (30).. In the initial stages of T2DM, there is an observed increase in the secretion of C-peptide. *In vitro* investigations suggest that excess C-peptide can accumulate within vessel walls. C-peptide instigates the chemotaxis of CD4(+) lymphocytes and monocytes in a concentration-dependent manner, thus encouraging their migration into the vessel wall. This chemotactic process is associated with pertussis toxin-sensitive G-proteins and a mechanism that is PI-3k-dependent (13, 14). C-peptide above physiological concentrations can stimulate the production of nitrite in mouse macrophages through the activation of the calcium/JAK2/STAT1 pathway (31).

When C-peptide is deficient, exogenous supplementation of C-peptide can play an anti-inflammatory role and prevent the apoptosis of endothelial cells. However, when C-peptide is higher than the physiological dose, excess C-peptide can promote inflammation by stimulating the chemotaxis of CD4(+) lymphocytes and monocytes, as well as promoting the pro-inflammatory role of macrophages.

## 3 C-peptide and diabetic kidney disease

DKD is acknowledged as a severe microvascular complication prompted by chronic hyperglycemia, resulting in structural and functional alterations in the kidney. Mitochondrial dysfunction leading to an elevated production of ROS and superoxide in states of high glucose is a principal initiating factor in the onset of diabetic complications (32). Excess ROS inhibition suppresses inhibition of mammalian rapamycin complex 1 (mTORC1), AMPK, and activation of the NF-κB, protein kinase C (PKC) pathway (33). mTORC1 and AMPK are central to the regulation of autophagy, which plays an important protective role in the kidney by preventing fibrosis and inflammation (34). PKC pathway activation affects vascular function by regulating endothelial permeability, vasoconstriction, extracellular matrix (ECM) maintenance, cell growth, angiogenesis, cytokine activation and leukocyte adhesion to influence vascular function (33). Activation of NF-κB further promotes the expression of inflammatory factors and adhesion molecules, triggering inflammation and fibrosis (35). On the one hand, elevated PKC induces endothelial-type nitric oxide synthase (eNOS) and increases nitric oxide (NO) utilization in early DKD (36). Increased NO contributes to vascular endothelial growth factor (VEGF) activation, which leads to endothelial dysfunction (37, 38). In addition, PKC can lead to activation of transforming growth factor-β (TGF-β) and plasminogen activator inhibitor 1 (PAI-1), resulting in fibronectin deposition, fine increased extracellular matrix deposition, causing glomerulosclerosis and renal fibrosis (36). A cross-sectional analysis conducted in Korea, which involved 1410 type 2 diabetes patients stratified based on quartiles of fasting C-peptide, revealed an increased prevalence of DKD in the group that fell within the highest quartile as compared to the lowest (OR= 2.65, 95% CI, (1.71, 4.12)) (39). Certain studies propose that C-peptide replacement therapy could represent a novel treatment approach for DKD. Several small clinical studies have suggested that short-term C-peptide supplementation in patients with T1DM can reduce eGFR and urinary protein excretion rates, without impacting glycemic and blood pressure control (40, 41). In 2000, a study of 21 normotensive patients with microalbuminuria found a significant reduction in urinary protein excretion rate following 3 months of combined insulin and C-peptide treatment, with no significant change in eGFR (42). In a subsequent cohort study of islet-kidney transplantation in T1DM, improved islet β-cell function post-transplantation was also found to enhance patient renal outcomes (43).A Meta-analysis (44) incorporating 4 human trials, and 18 animal trials, showed that DKD patients treated with C-peptide did not have a significant difference in GFR after treatment, but two studies reported a reduction in glomerular hyperfiltration. While, in diabetic rodent models, C-peptide led to a reduction in GFR reflecting a partial reduction in glomerular hyperfiltration.

Renal tubular cell membranes host G-protein coupled receptors (GPCR) with a high affinity for C-peptide (45). These peptides instigate a cascade of reactions upon binding to GPCR. In renal cells, the elements of signaling pathways influenced by C-peptide comprise Na^+^/K^+^/ATPase activity, endothelial calcium inward flow, PKC, ERK, c-Jun amino-terminal kinase (JNK), TGF-β/Smad, Inducible nitric oxide synthase (iNOS), Peroxisome proliferator-activated receptor (PPAR-γ), and PI-3k activity (46, 47).

It is suggested that C-peptide has the capacity to alleviate albuminuria and glomerular hyperfiltration by mitigating histological damage, which includes glomerular hypertrophy, glomerular thylakoid expansion, tubular interstitial inflammation, and tubular epithelial interstitial transformation. Furthermore, C-peptide is also believed to reduce tubular sodium reabsorption and glomerular hyperfiltration. Additionally, C-peptide can inhibit cellular apoptotic processes and tissue damage in renal tissues under high glucose conditions, thereby offering renal protection (46, 47).

The protective mechanisms of C-peptide on the kidney are mainly summarized as follows:

① Modulation of endothelial cell function to ameliorate hemodynamic disturbances

C-peptide replacement therapy was found to effectively rectify diabetic nephropathy in diabetic rat models, with nitric oxide (NO) likely serving as a key medium for the renal protective effects of C-peptide (10). C-peptide was observed to directly enter the nucleus of mesangial cells and inhibit the binding of NF-κB to p300 and the iNOS promoter, reducing the acetylation of histone H3K9ac, thereby repressing the expression of iNOS induced by NF-κB/p300 (48).

C-peptide demonstrated vasodilation capabilities for small arteries, and inhibited the Na^+^/K^+^/ATPase activity on renal tubules, lessening the reabsorption of sodium, thereby reducing the hyperfiltration induced by diabetes in the glomerulus (49).

② Inflammation and Fibrosis

In studies of type 1 diabetes mellitus (T1DM) mouse models, exogenous supplementation of C-peptide was found to aid in the reduction of pro-inflammatory cytokines such as IL17 and tumor necrosis factor-alpha (TNFα), and anti-inflammatory cytokines, like IL4 and IL10, in the urine (P <0.05). Additionally, an increase in the expression of the IL10 gene and a decrease in the expression of the TNFα gene were observed in the kidneys (50).

C-peptide also can alleviate renal fibrosis, thereby exerting renal protective effects. The activation of the TGFB1/SMAD3 signaling pathway can lead to the over-synthesis of type IV collagen protein (COL4), and deficiency in matrix metalloproteinases (MMP)-9 and MMP-2, which promote ECM deposition in renal cells. Islet transplantation showed a beneficial effect on the glomerular filtration membrane structure of early-stage diabetic nephropathy rats, reducing the thickness of the glomerular basement membrane, decreasing TGF-β1 and connective tissue growth factor (CTGF), increasing the expression of anti-fibrotic factors. This anti-fibrotic mechanism might be dependent on the restoration of C-peptide levels (51). Moreover, C-peptide was able to inhibit the expression of Col4 a1- a5 mRNA in the kidney, reducing COL4 and TGF-β1 protein levels, preventing the binding of SMAD3 to its sites in the promoters of Col4a1a2, Col4a3a4, and Col4a5, thereby repressing the production of COL4 (52). Finally, cell experiments showed that early C-peptide exerts a dual effect on MMP-9. As the observation time extended, C-peptide could induce an increase in MMP-9 expression, thereby reducing ECM accumulation and reversing DKD (53).

Although C-peptide replacement therapy significantly reduced the rate of urinary protein excretion in DKD in some early preclinical and clinical studies, the impact of C-peptide on eGFR remains contentious. Due to the small size, short duration, and lack of large-scale clinical trials, the evidence for C-peptide replacement therapy as a treatment for DKD remains limited within the scope of evidence-based medicine.

## 4 C-peptide and diabetic retinopathy

Chronic hyperglycemia incites a range of pathophysiological alterations within retinal cells. These alterations encompass an upsurge in the expression of matrix proteins such as collagen and fibronectin, thickening of the basement membrane, heightened retinal vascular permeability, and modifications in retinal blood flow (54). Beyond the potential for vision loss, diabetic retinopathy(DR) intensifies the risk of systemic vascular complications, thereby elevating the mortality rate among patients diagnosed with T2DM (55). Despite the role that the duration of diabetes and glycemic levels play in influencing DR (56), rigorous glycemic control does not entirely inhibit the evolution and progression of retinopathy.

Existing research on T1DM has revealed that residual fasting C-peptide exhibits protective qualities against the onset of DR (57). In a 2015 cross-sectional investigation involving 2062 patients with type 2 diabetes, it was demonstrated that C-peptide levels were inversely related to the prevalence of DR, irrespective of eGFR (39). Another cross-sectional study conducted in Shanghai, encompassing 4793 community-based patients with type 2 diabetes, reported that elevated fasting C-peptide levels acted as a protective factor against the prevalence of DR. It was observed that the prevalence of DR decreased as C-peptide levels increased (OR=0.73, 95% CI (0.62, 0.86), P<0.001) (58). Within the Veterans Affairs Diabetes Trial (VADT) (59), every increment of 1 pmol/mL in baseline C-peptide was correlated with a substantial 67.2% decrease in the prevalence of DR, coupled with a 47% reduction in the risk associated with DR progression. A prospective cohort study further demonstrated that patients who underwent islet transplantation experienced improved glycemic control and their progression of DR was considerably slower compared to those receiving intensive drug therapy (60). Moon et al. (61) managed to maintain C-peptide within the physiological concentration range for 16 weeks in diabetic mice by injecting ultra-long-lasting human C-peptide delivery into the vitreous body of the mice. The results indicated that maintaining the physiological concentration of C-peptide improved the formation of retinal neovascularization induced by high blood sugar in diabetic mice.

The protective role of C-peptide is attributed to the following mechanisms. Firstly, C-peptide enhances microvascular blood flow and improves microvascular endothelial function. It increases microvascular blood flow and mitigates vascular permeability by activating eNOS (62). It also counters ROS production by activating AMPK-α, which in turn inhibits VEGF’s increase in NADPH oxidase-dependent ROS production (63). C-peptide, along with ROS scavengers, diminishes VEGF-induced stress fiber formation that escalates vascular permeability (64). Moreover, C-peptide decreases the VEGF-induced breakdown of VE-cadherin, an endothelial cell-specific adhesion molecule responsible for connecting adjacent endothelial cells, thus maintaining endothelial cell integrity and reducing vascular permeability (65). Therefore, C-peptide may shield against retinopathy by inhibiting intracellular ROS production, reducing stress fiber formation, maintaining endothelial cell integrity, and decreasing VEGF-induced increases in microvascular permeability (11). High glucose conditions can trigger vascular leakage by activating TGase2 in the retina, but C-peptide can restrain this activation in the mouse retina, thereby decreasing vascular leakage (66).

Secondly, C-peptide also participate in regulating the composition of extracellular matrix proteins. Animal experiments have indicated that exogenous C-peptide supplementation can mitigate retinopathy by inhibiting the hyperglycemia-induced increase in fibronectin and reducing ECM deposition (67). Additionally, C-peptide exhibits insulin-mimetic properties, enhancing insulin action by increasing phosphorylation of insulin receptor substrate 1 and the activity of MAPK and PI-3k when insulin concentrations are low (68). Nevertheless, the current body of research on C-peptide replacement therapy in retinopathy is limited, and further investigation is warranted to explore its therapeutic effects.

## 5 C-peptide and diabetic macrovascular complications

The relationship between fasting C-peptide levels and macrovascular complications remains ambiguous, necessitating further investigation. In 2012, a cross-sectional study from Korea reported no association between C-peptide and macrovascular complications in T2DM patients (39). However, a contrasting result was obtained from a 2018 community-based T2DM study from Shanghai (58), which demonstrated that the prevalence of cardiovascular disease (CVD) escalated with increasing C-peptide levels. The risk of developing CVD correspondingly increased with rising C-peptide levels in a logistic regression analysis (OR = 1.27, 95% CI (1.13, 1.42), P<0.001).

In a cohort study (69)involving 2306 patients subjected to baseline coronary angiography and categorized according to the tertiles of C-peptide levels, the highest C-peptide group displayed an increased risk of all-cause mortality (HR = 1.46, 95% CI (1.15, 1.85), P = 0.002) and cardiac-cause mortality (HR = 1.58, 95% CI (1.15, 2.18), P = 0.002) when compared to the group with the lowest C-peptide levels. Additionally, the group with the highest C-peptide levels demonstrated elevated degrees of endothelial dysfunction, increased atherosclerotic markers, and more severe coronary artery lesions (69). A meta-analysis, including sixteen observational studies, eight cohort studies, and seven cross-sectional studies, showed the association between C-peptide and increased cardiovascular events was only observed in cross-sectional studies but not in cohort studies (70).

A study including 8 male T1DM,before and during dipyridamole administration using a randomized double-blind crossover protocol with infusion of C-peptide on two different days, suggested that short-term replacement of C-peptide improves myocardial function and myocardial perfusion (71). Endothelial cell damage and subsequent apoptosis in a state of high glucose are precipitated by oxidative stress, mitochondrial dysfunction, and abnormalities in calcium regulation (30). In addition, the adhesion and migration of monocytes beneath the endothelium are also among the key events in the early stages of atherosclerosis. In such an environment, C-peptide displays a protective effect on endothelial cells, mainly through the following mechanisms:

(1) Physiological concentrations of C-peptide can alleviate high glucose-mediated endothelial dysfunction, by reducing the expression of the cell surface protein VCAM-1 and decreasing the secretion of chemokines IL-8 and monocyte chemoattractant protein-1 (MCP-1) (72). However, this effect is not evident in environments with normal glucose levels; when C-peptide is in culture media with normal glucose levels, it does not significantly reduce the expression of VCAM-1 and chemokines (72). Furthermore, C-peptide combats high glucose-induced endothelial dysfunction by reducing NF-κB activation, potentially due to C-peptide induced phosphorylation of protein substrates in the cytoplasm or its interaction with NF-κB p65/p50 subunit, preventing DNA binding, impacting the expression of VCAM-1 and the secretion of MCP-1 and IL-8 (72, 73).

(2) C-peptide also exerts a protective effect by inhibiting the generation of ROS, reducing endothelial cell death, and preserving mitochondrial structure and function (46).

(3) In bovine and rat aortic endothelial cells, C-peptide demonstrates a concentration and time-dependent increase in endothelial NO release, which is induced by C-peptide through calcium internal mediation, thereby activating eNOS or ERK-1/-2 induced by C-peptide (74). In addition, studies have shown that C-peptide has inhibitory effects on endothelial cell proliferation in great saphenous vein bypass grafts. Insulin promotes neointima thickening, smooth muscle cell proliferation, and migration, while C-peptide can inhibit these effects (75).

These mechanisms collectively explain the protective effects of C-peptide on endothelial cells in a hyperglycemic environment.

In obese patients with type 2 diabetes, higher concentrations of C-peptide are associated with inflammation and exacerbation of the atherosclerotic process (30). In non-diabetic patients, it has been found that C-peptide levels are positively correlated with the incidence of several cardiovascular diseases, such as coronary artery disease and myocardial infarction (76). Conversely, in the early stages of T2DM, C-peptide secretion increases and can be deposited into the vascular endothelium, potentially promoting atherogenesis by initiating or facilitating the migration of monocytes to the endothelium (13). C-peptide can also induce lipid deposition and promote vascular smooth muscle cell (VSMC) recruitment and proliferation, all of which contribute to the development of atherosclerosis (13, 77). C-peptide can promote the transcriptional activity of PPAR-γ, as observed in renal tubular and lung injury, although most studies indicate that PPAR-γ has anti-inflammatory and anti-atherosclerotic effects, some research suggests that increased expression of PPAR-γ in macrophages can promote the occurrence of atherosclerosis, possibly related to the upregulation of CD36 expression in macrophages, which is deeply involved in the differentiation of monocytes into macrophages and the accumulation of oxidized LDL particles (78–81). In addition, C-peptide also can induce VSMC proliferation through the activation of SRC-kinase, PI-3K, and extracellular signal-regulated kinase 1/2 (82).

C-peptide plays different roles in T1DM, T2DM, or non-diabetic individuals, indicating that the regulation of atherosclerosis by C-peptide may be jointly influenced by blood glucose, insulin, and C-peptide concentrations. However, current research is not in-depth enough to further elucidate its mechanism. Therefore, further exploration of the regulatory effects of C-peptide, insulin, and the high-glucose environment on atherosclerosis is of significant importance.

## 6 C-peptide and diabetic peripheral neuropathy

A cross-sectional study enrolled 14,908 patients with T2DM showed that fasting C-peptide is negatively associated with diabetic peripheral neuropathy (DPN) (83). A predictive model that included 1278 T2DM patients showed that 2-hour postprandial C-peptide/fasting C-peptide was a protective factor for DPN (84). In a type 1 diabetic rat model, C-peptide replacement therapy has been observed to prevent the development of deficits in nerve conduction velocity (NCV) and alleviate both acute and chronic DPN as T1DM naturally progresses (12). Furthermore, in BB/Wor rats characterized by reduced intraneural perfusion and increased oxidative stress, C-peptide also prevents neurovascular defects and decreases thermal nociceptive hypersensitivity (85). The mechanism by which C-peptide ameliorates neuropathy might involve the stimulation of the NO system by C-peptide, acting directly on nerve fibers or mediating vasodilation via NO, rather than by improving oxidative stress (73, 85).

The Na^+^/K^+^/ATPase, a prevalent membrane enzyme, exhibits reduced activity in peripheral nerves, leading to Na+ channel inactivation and myelin swelling (12). In BB/Wor rats, two consecutive months of C-peptide replacement treatment diminished myelin swelling and improved neural Na^+^/K^+^/ATPase defects (12). Thus, current findings indicate that in a T1DM mouse model, physiological concentrations of C-peptide can provide neuroprotective functions by enhancing neuroperfusion through NO-sensitive neurovascular mechanisms and ameliorating Na^+^/K^+^/ATPase deficits.

In a 2003 clinical trial conducted by Ekberg et al. (86), which included 46 patients, C-peptide replacement therapy for three months significantly improved sensory nerve conduction velocity (SCV) in the C-peptide treatment group, along with enhanced vibrotactile sensation, although no temperature and motor nerve conduction velocity (MCV) improvements were recorded. In 2007, the study scope was broadened to incorporate a six-month period of C-peptide replacement therapy and to include a population of T1DM patients exhibiting DPN symptoms. Findings indicated that C-peptide replacement therapy significantly improved SCV among patients displaying DPN symptoms, particularly noticeable in those with less compromised neurological function at baseline (87). In 2016, a clinical study that included 250 patients with DPN who received long-acting C-peptide (2.4 mg, 0.8 mg) weekly to DPN for 52 weeks, found significant improvements in patients’ vibration perception threshold (VPT). However, sural nerve conduction velocity (SNCV), other electrophysiological variables, or modified Toronto Clinical Neuropathy Score (mTCNS) were not significantly different from placebo control patients (88). It is worth noting that, the C-peptide of patients increased to 1.8-2.2 nmol/L (low dose) and 5.6-6.8 nmol/L (high dose), which is higher than the physiological concentration of C-peptide. As previously articulated, the infusion of ultra-long-lasting human C-peptide into the vitreous body of mice, maintaining C-peptide within the physiological concentration range, yielded favorable therapeutic outcomes. In this clinical investigation, the failure to achieve significant improvement in SNCV, other electrophysiological variables, or mTCNS in DPN patients may be attributed to the elevated C-peptide concentrations, which exceeded physiological levels. This observation leads us to conjecture that the optimal therapeutic effect may lie within a specific concentration range. Consequently, we propose that future basic and clinical research into C-peptide replacement therapies should focus on the meticulous monitoring of post-treatment C-peptide levels. Additionally, these studies should explore the optimal range for maintaining both fast C-peptide and post-radical C-peptide concentrations, as this may be pivotal in enhancing the efficacy of the treatment.

In T2DM rats, sensory NCV slowing or nociceptive hyperalgesia was only observed with reduced neurovascular perfusion and was not accompanied by increased oxidative stress, and C-peptide replacement therapy did not improve neuropathy (85). A study in a community-based population (89) discovered a negative association between C-peptide levels and the development of diabetic peripheral neuropathy in a T2DM population. Given the disparate mechanisms underpinning DPN in T1DM and T2DM, it is noteworthy that the present study specifically demonstrated no significant amelioration of DPN in T2DM patients via C-peptide replacement. This underscores the need for further investigation into the association between overproduction of C-peptide and DPN in T2DM patients, necessitating additional animal experiments.

## 7 C-peptide and diabetic emergencies

Diabetic emergencies include diabetic ketoacidosis (DKA), hyperglycaemic hyperosmolar state (HHS) and hypoglycemia (90). Serum C-peptide is negatively correlated with the risk of DKA (91) and hypoglycemia (92), but there is currently no research exploring the relationship between C-peptide and HHS. A retrospective study (91) involving 234 children and adolescents with T1DM, based on serum C-peptide levels at diagnosis and 15 years post-diagnosis, compared laboratory results and diabetes complication incidence between the two groups after 15 years. Results showed that patients with higher C-peptide levels (after 15 years) used lower doses of insulin, and in the group with lower C-peptide levels, the incidence rate of DKA was higher, consistent with the conclusions of the previous studies (93, 94). A study (95) based on clinical and biochemical characteristics to predict the risk of diabetic ketosis (DK) in patients with T2DM shows that 2-hour postprandial C-peptide levels are negatively correlated with the risk of diabetic ketosis. However, the number of relevant studies is relatively small, and more clinical studies are needed to support the relevant conclusions. Additionally, whether C-peptide replacement therapy has a preventive effect on ketoacidosis poisoning remains to be further researched.

A study involving 1565 patients with T2DM from the Veterans Affairs Diabetes Trial showed that measuring fasting C-peptide levels at baseline was negatively correlated with the risk of severe hypoglycemia (96). Zenz et al. (97) found that under hypoglycemic conditions, retained β-cell function (C-peptide positive) may help to regulate blood glucose levels more effectively. C-peptide positive patients have higher glucagon concentrations and endogenous glucose production (EGP) during hypoglycemia, suggesting better response mechanisms under hypoglycemic conditions Hope et al. (98) conducted continuous glucose monitoring on 17 insulin-treated T2DM patients and matched controls, and surveyed 256 insulin-treated T2DM patients and 209 T1DM patients. The results showed that patients with lower random C-peptide levels (rCP <200 pmol/l) were more prone to hypoglycemia, with more frequent and prolonged episodes. Patients with preserved C-peptide had fewer hypoglycemic events at night. This finding suggests that low rCP levels can serve as a practical, stable, and economical biomarker for hypoglycemia risk assessment, aiding the management and treatment of T2DM. C-peptide may protect against hypoglycemia by increasing α-cell response to low blood sugar and promoting glucagon secretion (99). In adult T1DM patients, β-cell responsiveness to hyperglycemia and α-cell responsiveness to hypoglycemia were only observed when residual C-peptide levels were higher. Residual C-peptide may assist in blood glucose control, and a clinical trial in Japan also showed that C-peptide is independently associated with glucagon levels (100). Moore et al. (101) explored the role of C-peptide in insulin-induced hypoglycemia by testing the effects of C-peptide infusion on glucagon secretion under isoglycemic and hypoglycemic conditions in dogs (5 males/4 females). In the experiments, glucagon secretion remained unchanged in the isoglycemic-hyperinsulinemic response in the C-peptide infusion group, whereas it increased twofold during hypoglycemia. These data suggest that the presence of C-peptide maintains glucagon secretion during isoglycemia and enhances it during hypoglycemia, which explains why T1DM patients with residual insulin secretory capacity are less susceptible to hypoglycemia. However, the regulatory mechanism of C-peptide on glucagon is yet to be reported. Lower C-peptide levels have been observed to be associated with greater glucose fluctuation and higher hypoglycemia risk, leading to attention to its p otential vital role in blood sugar stability regulation. Although C-peptide, as a byproduct of insulin synthesis, has not been fully revealed in terms of its biological functions in diabetes treatment and metabolic regulation, more and more experimental data support that the maintenance and stability of C-peptide levels are important in blood sugar control and diabetes-related complications prevention. Therefore, C-peptide could be considered an important clinical target for glucose control in diabetes treatment.

## 8 Conclusion

C-peptide, a bioactive peptide with a plethora of functionalities, has a profound biological significance. It exhibits antioxidant, anti-apoptotic, and anti-inflammatory effects primarily through binding with cellular surface signaling molecules to activate downstream pathways or regulating intracellular transcriptional processes.

**Figure 1:**
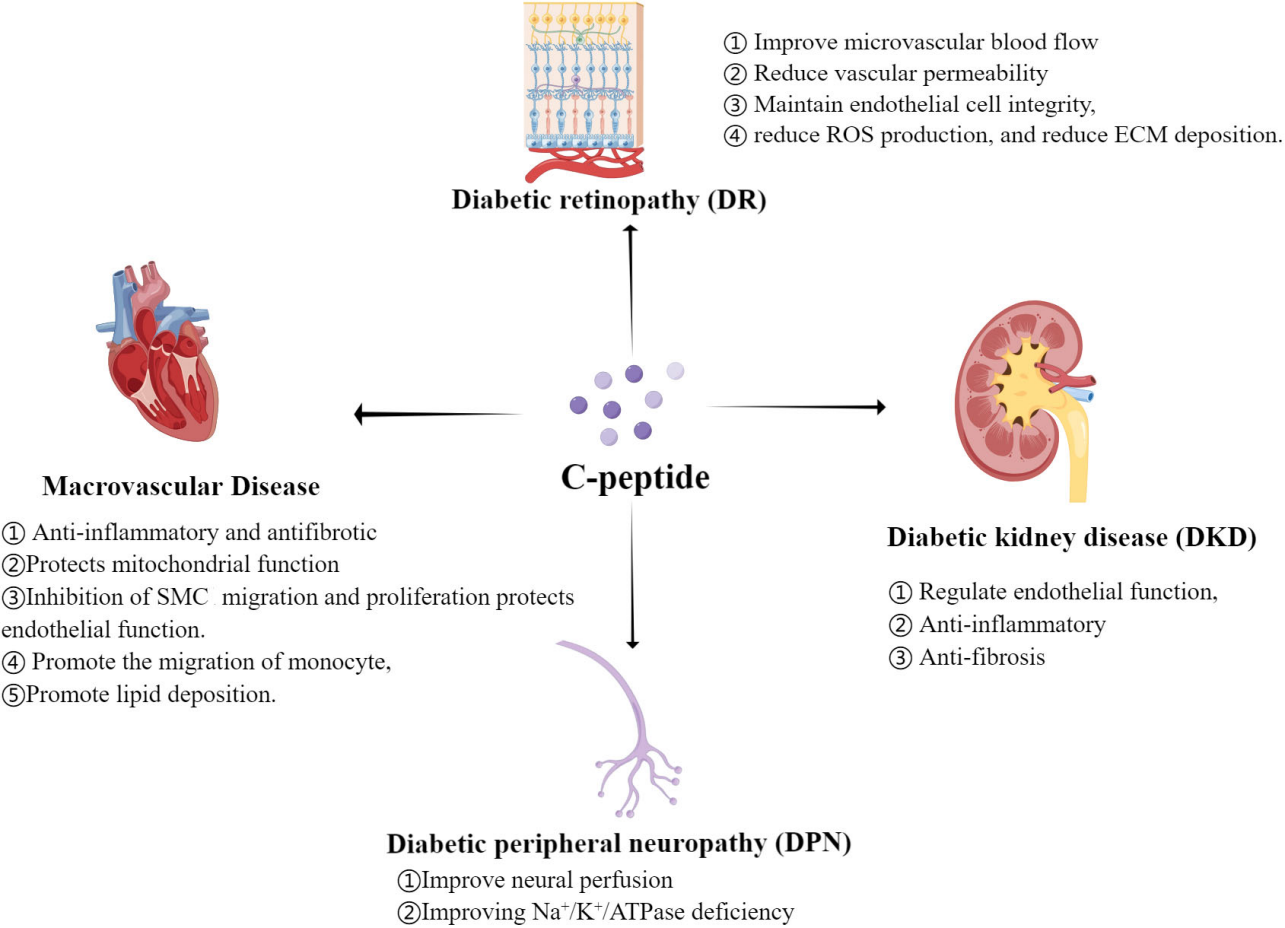
Regulation mechanism of C-peptide on Diabetic Chronic Complications.(By Figdraw.) ROS, reactive oxygen species; ECM, extracellular matrix; SMC, smooth muscle cell.

The complex pattern of the relationship between C-peptide and diabetic chronic complications (**Figure 1**) has not yet been fully understood. The underlying mechanisms might be associated with: 1) dose-dependent effects of C-peptide; 2) varied affinities with different receptors under physiological and pathological conditions; 3) divergent responses in different cells and tissues; and 4) potential interactions with other molecules altering C-peptide’s effects. Future basic and clinical studies of C-peptide replacement therapies will need to focus on baseline levels of C-peptide in addition to more attention also needs to be paid to post-treatment C-peptide levels to explore the optimal range of fasting C-peptide and postprandial C-peptide maintenance.

In conclusion, a deeper understanding of the role of C-peptide in the pathogenesis of diabetic complications could be key to their prevention and treatment. This would not only help to clarify the differences in the mechanisms underlying the onset of complications in type 1 and type 2 diabetes but also provide practical implications for clinical treatments.

## Author contributions

JChen: Writing – original draft, Writing – review & editing. YH: Writing – original draft, Writing – review & editing. CL: Writing – original draft. JChi: Writing – review & editing. YW: Writing – review & editing. LX: Writing – original draft, Writing – review & editing.
